# LncRNA-FAM66C Was Identified as a Key Regulator for Modulating Tumor Microenvironment and Hypoxia-Related Pathways in Glioblastoma

**DOI:** 10.3389/fpubh.2022.898270

**Published:** 2022-07-06

**Authors:** Dan Liu, Yue Wan, Ning Qu, Qiang Fu, Chao Liang, Lingda Zeng, Yang Yang

**Affiliations:** ^1^Oncology Department, Jinzhou Central Hospital, Jinzhou, China; ^2^Department of Pediatrics, Jinzhou Central Hospital, Jinzhou, China; ^3^Department of Neurosurgery, Shengjing Hospital Affiliated to China Medical University, Shenyang, China; ^4^Department of General Surgery, The First Affiliated Hospital of Jinzhou Medical University, Jinzhou, China; ^5^Department of Otorhinolaryngology Surgery, Jinzhou Central Hospital, Jinzhou, China; ^6^Department of Neurosurgery, Jinzhou Central Hospital, Jinzhou, China

**Keywords:** hypoxia, long non-coding RNAs, molecular subtypes, tumor microenvironment, transcription factors, FAM66C, biomarkers, bioinformatics analysis

## Abstract

Although the role of hypoxia has been greatly explored and unveiled in glioblastoma (GBM), the mechanism of hypoxia-related long non-coding (lnc) RNAs has not been clearly understood. This study aims to reveal the crosstalk among hypoxia-related lncRNAs, tumor microenvironment (TME), and tumorigenesis for GBM. Gene expression profiles of GBM patients were used as a basis for identifying hypoxia-related lncRNAs. Unsupervised consensus clustering was conducted for classifying samples into different molecular subtypes. Gene set enrichment analysis (GSEA) was performed to analyze the enrichment of a series of genes or gene signatures. Three molecular subtypes were constructed based on eight identified hypoxia-related lncRNAs. Oncogenic pathways, such as epithelial mesenchymal transition (EMT), tumor necrosis factor-α (TNF-α) signaling, angiogenesis, hypoxia, P53 signaling, and glycolysis pathways, were significantly enriched in C1 subtype with poor overall survival. C1 subtype showed high immune infiltration and high expression of immune checkpoints. Furthermore, we identified 10 transcription factors (TFs) that were highly correlated with lncRNA-FAM66C. Three key lncRNAs (ADAMTS9-AS2, LINC00968, and LUCAT1) were screened as prognostic biomarkers for GBM. This study shed light on the important role of hypoxia-related lncRNAs for TME modulation and tumorigenesis in GBM. The eight identified hypoxia-related lncRNAs, especially FAM66C may serve as key regulators involving in hypoxia-related pathways.

## Introduction

Glioblastoma (GBM) is the most diagnosed cancer type in malignant brain tumors, contributing a proportion of about 57% within gliomas. Age-adjusted incidence of primary GBM varied greatly depending countries from 0.51 to 3.21 per 100,000, with a diagnosed peak in older individuals at the ages between 75 and 79 (1, 2). Genders and race make an effect on the incidence as well. In the United States, the male-to-female ratio is 4.00 vs. 2.53 per 100,000, and non-Hispanic whites have the highest incidence (2). Due to the complex diagnosis and resistance or poor sensitivity of the brain to chemotherapeutic molecules, GBM presents dismal prognosis with median overall survival (OS) of 14.4 months (3). Therefore, understanding the molecular mechanisms underlying its aggressive behavior may benefit management and targeted therapies for GBM patients.

Molecular features, such as isocitrate dehydrogenase (IDH) mutations, TERT promoter mutations, MGMT promoter methylation, and chromosome 1p/19q co-deletion status, have been found to serve as indicators for glioma prognosis and classifications (4, 5). World Health Organization (WHO) proposes three molecular classifications for GBM, IDH wild-type (wt) (corresponding to primary GBM), IDH-mutant (corresponding to secondary GBM), and NOS (lacking any access to molecular diagnostic testing) (6). Different molecular features lead to different outcomes or sensitivity to chemotherapy, which improves accurate treatments for GBM patients. For example, IDH-mutant status is associated with longer OS after chemoradiotherapy comparing with wt IDH (7). Besides chemotherapy and radiotherapy, other strategies, such as cancer vaccines, oncolytic viral therapies, immune checkpoint blockade, and chimeric antigen receptor T-cell therapy, have been developing, and clinical trials are undergoing (8).

Profound understanding of GBM pathogenesis is a basis for the targeted therapies. Abundant studies have demonstrated that hypoxia is a critical hallmark of cancer development, not excluding GBM (9, 10). Rapid tumor cell proliferation accompanying with an erratic tumor neovascularization leads to unfavorable oxygen diffusion and thus affects the formation of tumor microenvironment (TME) (11). Hypoxia inducible factor (HIF) is a transcription factor (TF) mainly for regulating hypoxic metabolism, whose upregulation is associated with worse survival (12, 13). In the regulation of HIF, long non-coding RNAs (lncRNAs) serve as activators or suppressors for interfering HIF pathway (14, 15). LncRNAs are recognized as important regulators serving as onco- and tumor-suppressor lncRNAs on the activity of TFs, RNA-binding proteins, and microRNAs. The dysregulation of lncRNAs can alter cancer genes and cancer cell proliferation synergistically with dysregulated cancer pathways (16).

A number of studies have explored a series of lncRNAs serving as biomarkers for GBM, such as HIF1A-AS2 (17), HOTAIR (18), and HOXA11-AS (19). However, a comprehensive study on the relation between hypoxia and lncRNAs in GBM has not been unveiled yet.

In this study, we employed GBM expression profiles from public databases to explore the role of lncRNAs in hypoxia and tumor progression. Using hypoxia-related lncRNAs, we constructed three molecular subtypes for comprehensively characterizing the link of lncRNAs in orchestrating TME and controlling tumorigenesis. We found that many oncogenic pathways and immune-related pathways linked to hypoxia-related lncRNAs. Unlike with some other cancer types, repeat tissue sampling is more complicated and risky in GBM patients. Therefore, effective biomarkers for predicting efficacy and prognosis before and after treatment are substantially needed. The study also identified three key hypoxia-related lncRNAs for potentially applying in GBM patients.

## Materials and Methods

### Data Information

The workflow of this study was shown in Supplementary Figure S1. Using GDC Application Programming Interface (API), we downloaded RNA-seq data of GBM from The Cancer Genome Atlas (TCGA) database (https://portal.gdc.cancer.gov/), named as TCGA-GBM dataset. “mRNAseq_693 (batch 1)” and “mRNAseq_325 (batch 2)” datasets were downloaded from Chinese Glioma Genome Atlas (CGGA) database (http://www.cgga.org.cn/), and they were combined through the function “combat” in sva R package (20), named as CGGA-GBM dataset. TCGA-GBM and CGGA-GBM samples (only primary GBM samples were included) with clinical information were retained (total 151 samples and 218 samples, respectively). GSE108474 dataset was downloaded from Gene Expression Omnibus (GEO) database (https://www.ncbi.nlm.nih.gov/geo/).

### Identification of Hypoxia-Related LncRNAs

Hypoxia-related genes were obtained from “HALLMARK_HYPOXIA” in MSigDB (https://www.gsea-msigdb.org/gsea/msigdb/). GTF file (GRCh38.p13) was obtained from GENCODE (https://www.gencodegenes.org/) for dividing the expression profiles into mRNA and lncRNA. First, expression of a gene set in hypoxia pathway was used as an input to calculate hypoxia score for each sample in TCGA-GBM and CGGA-GBM datasets through single sample GSEA (ssGSEA) in GSVA R package (21). Then, Pearson correlation analysis was applied to assess the correlation between hypoxia score and lncRNA expression in two datasets, respectively. |coefficient| > 0.4 and *p* < 0.05 were determined to screen lncRNAs, defined as hypoxia-related lncRNAs.

### Unsupervised Consensus Clustering

Unsupervised consensus clustering in ConsensusClusterPlus R package was implemented to construct molecular subtypes based on the expression of hypoxia-related lncRNAs (22). PAM and “canberra” algorithms were selected as measure distance. In total, 500 bootstraps were conducted with each has 80% samples of training cohort. Consensus matrix and cumulative distribution function (CDF) were used to confirm the optimal cluster number k (set from 2 to 10).

### Assessment of Genomic Features and Gene Mutations

The scores of genomic features including aneuploidy, homologous recombination defects, fraction altered (the fraction of bases pairs deviating from the baseline ploidy), number of segments, and tumor mutation burden (non-silent mutation rate) were obtained from the previous research (23). The top 20 mutated genes were called by mutect2 tool (Fisher's exact test, *p* < 0.05) (24).

### Gene Set Enrichment Analysis

Gene set enrichment analysis is a statistical method for evaluating the enrichment of a gene set in a list of gene signatures ranked by interested phenotypes, such as a pathway with a bulk of genes (25). We used GSEA to analyze all hallmark pathways and identify the differentially enriched hallmark pathways among different molecular subtypes. All hallmark pathways were collected from MSigDB (v7.4, https://www.gsea-msigdb.org/gsea/msigdb/) (26). False discovery rate (FDR) < 0.05 was selected to screen significantly enriched pathways. GSEA was also performed in TME analysis including the fraction of immune cells and the enrichment of immune checkpoints. The gene signatures of immune cells and immune checkpoints were obtained from Senbabaoglu et al. and HisgAtlas database, respectively (27, 28). ANOVA was performed among three subtypes.

### Estimation of Stromal and Immune Cells in Malignant Tumors Using Expression Data

Estimation of stromal and immune cells in malignant tumors using expression data (ESTIMATE) method is conducted for evaluating the immune infiltration based on a series of gene signatures through ssGSEA (29). It is a popular tool for comparing the enrichment of stromal cells and immune cells between tumor samples and normal samples. Immune score, stromal score, and ESTIMATE score were calculated, where ESTIMATE score is the combined score of immune score and stromal score. ANOVA was conducted among three subtypes.

### Tumor Immune Dysfunction and Exclusion Analysis

Tumor immune dysfunction and exclusion (TIDE) analysis (http://tide.dfci.harvard.edu) was used to calculate the enrichment score of three immunosuppressive cells, including myeloid-derived suppressor cells (MDSCs), M2 tumor-associated macrophages (TAMs), and cancer-associated fibroblasts (CAFs) (30). TIDE describes tumor immune escape through characterizing T-cell dysfunction and T-cell exclusion based on identified gene signatures, which can predict the sensitivity to immune checkpoint blockade. The Kruskal–Wallis test was conducted among three subtypes.

### Analysis of the Relation Between LncRNAs and TFs

Transcription factor activity was calculated according to the algorithm of Garcia-Alonso et al. (31), and ANOVA was conducted to screen differential TFs among three subtypes (*p* < 0.05). Pearson correlation analysis was performed to assess the relation between nuclear lncRNAs and differential TFs (|R| ≥ 0.3, *p* < 0.05). ClusterProfiler R package was used to annotate significantly enriched pathways of upregulated TFs (32). The top 10 enriched pathways were visualized (*p* < 0.05).

### First-Order Partial Correlation Analysis

First-order partial correlation analysis can eliminate the effect of one variable when there are criterion variable and two predictor variables, which is able to identify meaningful gene–gene associations (33). The association between the two variables may be sharply reduced after removing the effect of the third variable. It was applied to identify key lncRNAs linking hypoxia-related genes to hypoxia score. The hypoxia score was assumed to be *x*, and hypoxia-associated gene expression was *y*. The first-order partial correlation between *x* and *y* conditioned on lncRNAs was:


$$\begin{array}{l} r_{xylncRNA}=\frac{r_{xy}-r_{xlncRNA}^{*}r_{ylncRNA}}{\sqrt{{\left(1-r_{xlncRNA}^{2}\right)}^{*}\left(1-r_{ylncRNA}^{2}\;\right)}} \end{array}$$

### Construction of a Prognostic Model Based on Key LncRNAs

Three identified key hypoxia-related lncRNAs were included as three indictors to establish a prognostic model defining as: risk score = Σ (beta i × Exp i). Beta represents the coefficients of univariate Cox regression. Exp represents lncRNA expression and i represents lncRNAs. Median of risk score was determined as a cut-off to classify samples into high-risk and low-risk groups.

### Statistical Analysis

Statistical analysis was conducted in the R software (4.1.0). Default parameters were used if there was no indication. Statistical methods were described in the corresponding sections. *p* < 0.05 was considered as significant.

## Results

### Hypoxia-Related LncRNAs Are Associated With Hypoxia Score and Survival

**Figure 1:**
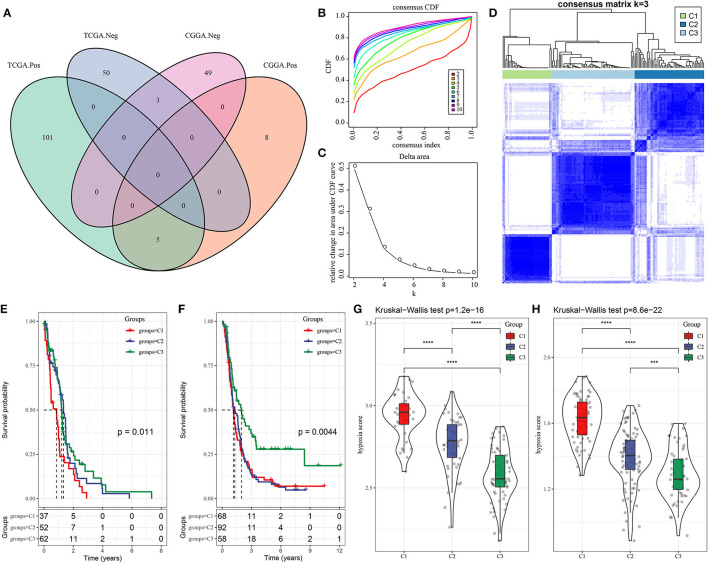
Three molecular subtypes of GBM based on hypoxia-related long non-coding RNAs (lncRNAs). **(A)** Venn plot of hypoxia-related lncRNA significantly associated with hypoxia score in TCGA-GBM and CGGA-GBM datasets. Pos and Neg represent positive and negative correlations between lncRNAs and hypoxia score. **(B,C)** CDF curve and relative change in area under CDF curve when cluster number *k* = 2–10. **(D)** Consensus matrix when *k* = 3. **(E,F)** Kaplan–Meier survival plot of three subtypes in TCGA-GBM and CGGA-GBM datasets. Log-rank test was conducted. **(G,H)** Hypoxia score of three subtypes in TCGA-GBM and CGGA-GBM datasets. The Kruskal–Wallis test was conducted. Log-rank test was conducted in **(E,F)**. The Kruskal-Wallis test was conducted in **(G,H)**. ***P < 0.001, ****p < 0.0001. GBM, glioblastoma; TCGA, The Cancer Genome Atlas; CGGA, Chinese Glioma Genome Atlas; CDF, cumulative distribution function.

Hypoxia is a common characteristic of tumors that has been linked to poor survival and invasive traits in a variety of cancers, including GBM. We aimed to build a molecular subtyping system focusing on hypoxia-related lncRNAs to better understand the function of hypoxia in characterizing TME. To begin, hypoxia-related lncRNAs from the hypoxia hallmark pathway were screened using the Pearson correlation analysis to find lncRNAs that were strongly connected with hypoxia enrichment score. In TCGA-GBM and CGGA-GBM datasets, 159 and 65 lncRNAs were screened, respectively, with an intersection of eight lncRNAs between two datasets including AC017002.1, ADAMTS9-AS2, LINC00968, LUCAT1, MIR210HG, AC114730.3, FAM66C, and MYCNOS (Supplementary Table S1; Figure 1A). Then, unsupervised consensus clustering was applied based on the eight lncRNAs in TCGA-GBM dataset. The optimal cluster number *k* = 3 was determined by CDF, relative change in area under CDF curve and consensus matrix (Figures 1B–D). As a result, three molecular subtypes of C1, C2, and C3 were defined to classify GBM samples. Not surprisingly, C3 subtype with the lowest hypoxia score had the longest survival, whereas C1 subtype with the highest hypoxia score had the worst survival in both TCGA-GBM and CGGA-GBM datasets (Figures 1E–H), indicating that the eight hypoxia-related lncRNAs did have a strong correlation with GBM progression.

### C3 Subtype Has the Highest Proportion of IDH-Mutant Type

**Figure 2:**
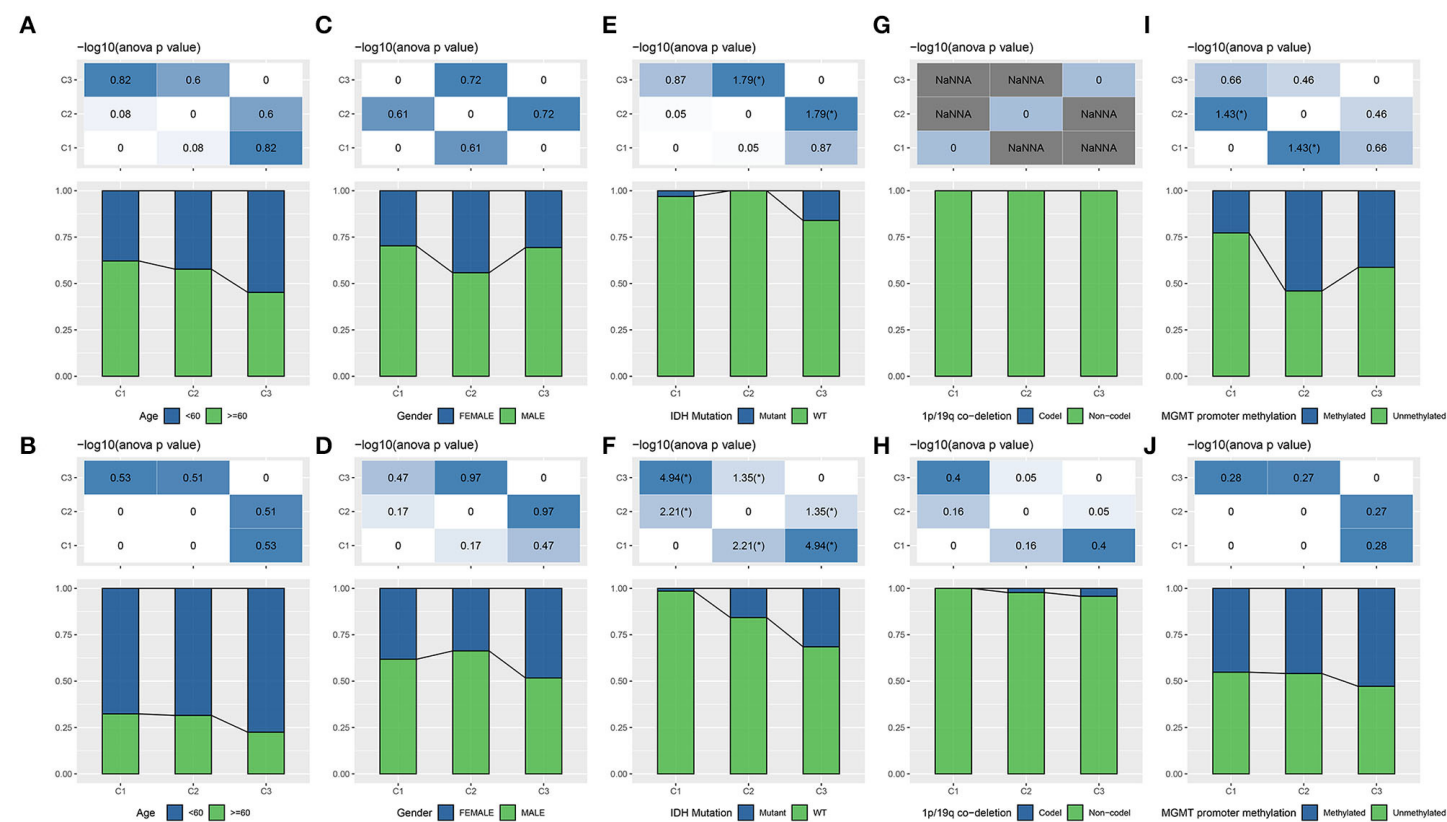
The distribution of different clinical features including age **(A,B)**, gender **(C,D)**, and molecular features including IDH **(E,F)**, 1p/19q co-deletion **(G,H)**, MGMT promoter methylation **(I,J)** in three subtypes. The upper group is TCGA-GBM dataset and the lower group is CGGA-GBM dataset. ANOVA was conducted. *p < 0.05. NaNNA, no available data. IDH, isocitrate dehydrogenase; GBM, glioblastoma; TCGA, The Cancer Genome Atlas; CGGA, Chinese Glioma Genome Atlas.

Age and gender were reported to be associated with GBM occurrence and survival, with that advanced ages had a higher incidence and poor survival rate (34). Males are more common to develop GBM comparing with females. We investigated the distribution of three subtypes in different ages and genders. Although no significant difference was observed, still a tendency indicated that older ages had a higher proportion in subtypes with worse survival in both two datasets (Figures 2A,B). Of genders, all three subtypes presented higher proportions of males, but no difference and regularity were observed (Figures 2C,D).

Isocitrate dehydrogenase, 1p/19q, and MGMT promoter methylation are three important features reported to serve as prognostic indicators for gliomas (5), with IDH-wt and IDH-mutant are two WHO classifications for GBM (6). To illustrate whether a correlation was shown between these molecular features and lncRNA expression patterns, we analyzed the distribution of three features in three subtypes. As a result, we found that C3 subtype had the highest proportion of the combination of IDH-mutant, 1p/19q co-deletion, and MGMT promoter methylation (Figures 2E–J), which was consistent with the previous observations that these three status were associated with longer survival (4, 5). Especially, a higher proportion with over than 1/4 of samples in C3 subtype had IDH mutation in CGGA-GBM dataset (*p* < 0.05, Figure 2F). IDH mutation has been demonstrated to have an effect on cellular metabolism, cancer biology and oncogenesis (35). The above results supported that the molecular subtyping based on the eight lncRNAs had a close relation with clinical features and classical molecular features.

### Different Mutation Patterns Among Three Subtypes

Furthermore, we tried to elucidate if there was a difference of genomic features among three subtypes. Five aspects, including aneuploidy, homologous recombination defects, fraction altered (the fraction of bases pairs present in the copy number profiles deviating from the baseline ploidy), number of segments (the total number of segments present in the copy number profile), and tumor mutation burden, were selected, and corresponding scores of three subtypes were calculated in TCGA-GBM dataset (Supplementary Figure S2A). Only homologous recombination defects and number of segments were shown to be differential among three subtypes, with a tendency of samples with worse survival had lower scores. Furthermore, analysis on the correlation between genomic features and hypoxia score also revealed a negative correlation of homologous recombination defects and number of segments with hypoxia score (Supplementary Figure S2B, *R* = −0.318, *p* = 0.000114; *R* = −0.396, *p* = 8.34e−07, respectively), which was accordant with the result that C3 subtype with the longest survival had the lowest hypoxia score (Figures 1E–H).

In addition, we analyzed gene mutations of all samples in TCGA-GBM dataset and screened the top 20 significantly mutated genes. Overall, C3 subtype had the most mutations and copy number variations (Supplementary Figure S2C). In C1 subtype, *CKMT1A* and *MKS1* were mostly mutated with frequencies of 14 and 11%, respectively. In C2 subtype, *DNAH11* and *PUM1* consisted of 19 and 15% mutation rate, respectively. In C3 subtype, *PIK3CA* mutations contributed a highest proportion of 21%. Notably, *IDH1* was only found to be mutated in C3 subtype, and the majority mutation type was missense, which was consistent with the previous research that IDH-mutant patients had longer OS (35). Three subtypes manifested the distinct mutation patterns that may contribute to distinct prognosis.

### Oncogenic Pathways Are Highly Enriched in C1 Subtype

**Figure 3:**
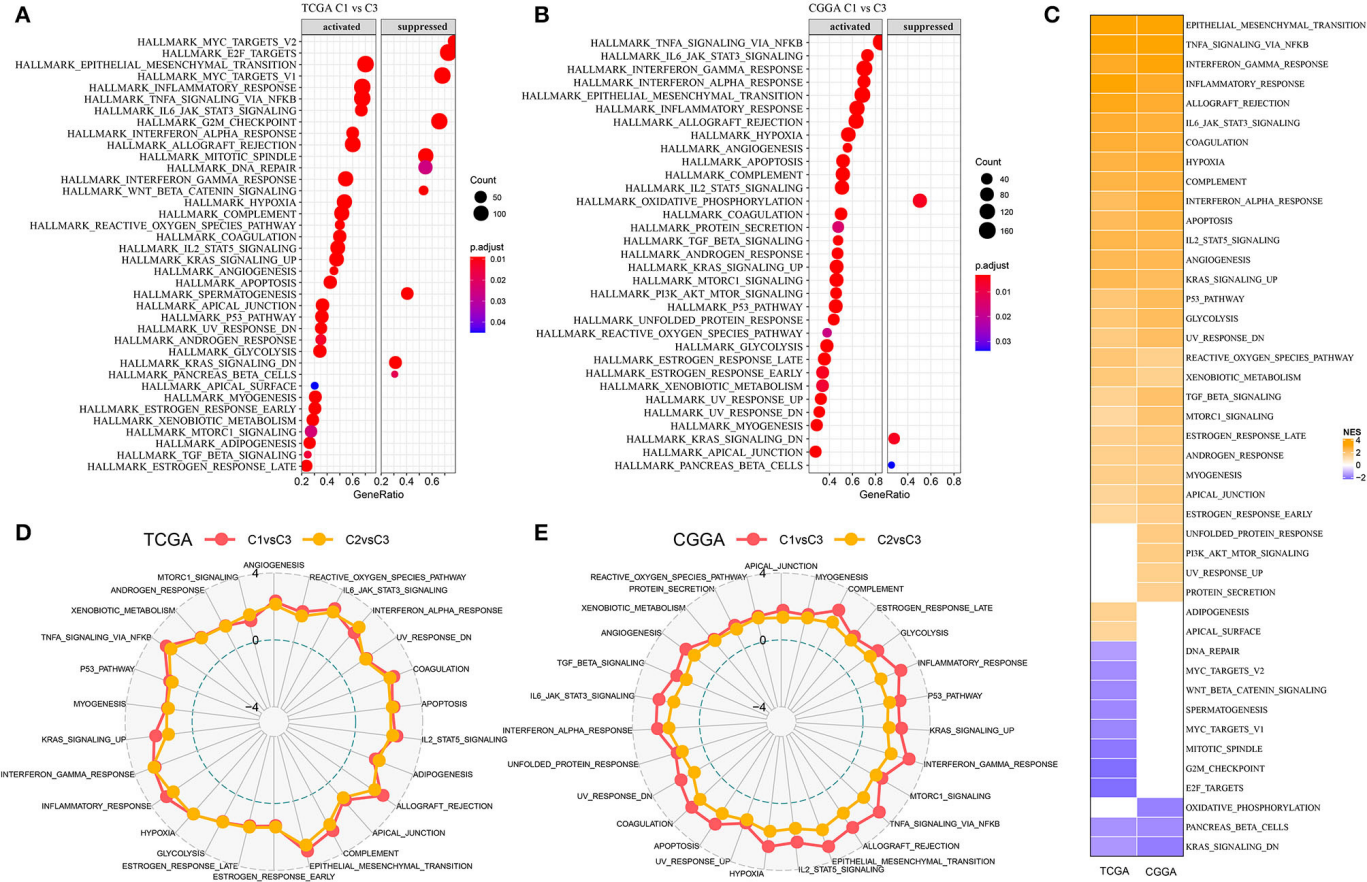
Differentially enriched pathways among three subtypes. **(A,B)** Relatively activated and suppressed hallmark pathways of C1 subtype comparing with C3 subtype in TCGA-GBM and CGGA-GBM datasets. Count represents the number of genes. **(C)** Normalized enriched score (NES) of differentially enriched pathways in two datasets. **(D,E)** Rader plots of upregulated pathways of C1 vs. C3 and C2 vs. C3 in two datasets. Dotted circles from inner to outside indicate NES of −4, 0, and 4, respectively.

As three subtypes based on lncRNA expression have been demonstrated to be associated with survival and molecular features, we assumed that these eight lncRNAs may be involved in critical pathways affecting prognosis. By using GSEA on hallmark pathways for samples in two datasets, significantly enriched pathways were outputted (FDR < 0.05, Figures 3A,B). In TCGA-GBM dataset, a total of 38 pathways were dysregulated with 28 activated and 10 suppressed, comparing with C3 subtype (Figure 3A). In CGGA-GBM dataset, 30 pathways were activated, and three pathways were suppressed in C1 subtype in comparison with C3 subtype. Within these activated pathways, oncogenic pathways were highly enriched such as epithelial mesenchymal transition (EMT), tumor necrosis factor-α (TNF-α) signaling *via* NFKB, hypoxia, angiogenesis, KRAS signaling, P53 signaling, and glycolysis pathways (Figure 3C). The above-enriched pathways have been illustrated to be highly activated in cancer, and they are commonly activated together with hypoxia such as EMT and angiogenesis can be induced by hypoxia (36, 37). Immune-related pathways were also enriched such as interferon-γ response, inflammatory response, and IL6-JAK/STAT3 signaling pathways. In comparison with C2 subtype, these activated pathways were more enriched in C1 subtype (Figures 3D,E), implying that the eight lncRNAs for subtyping possibly served as important regulators in activating these oncogenic or immune-related pathways.

### Worse Survival Is Associated With Higher Immune Infiltration

**Figure 4:**
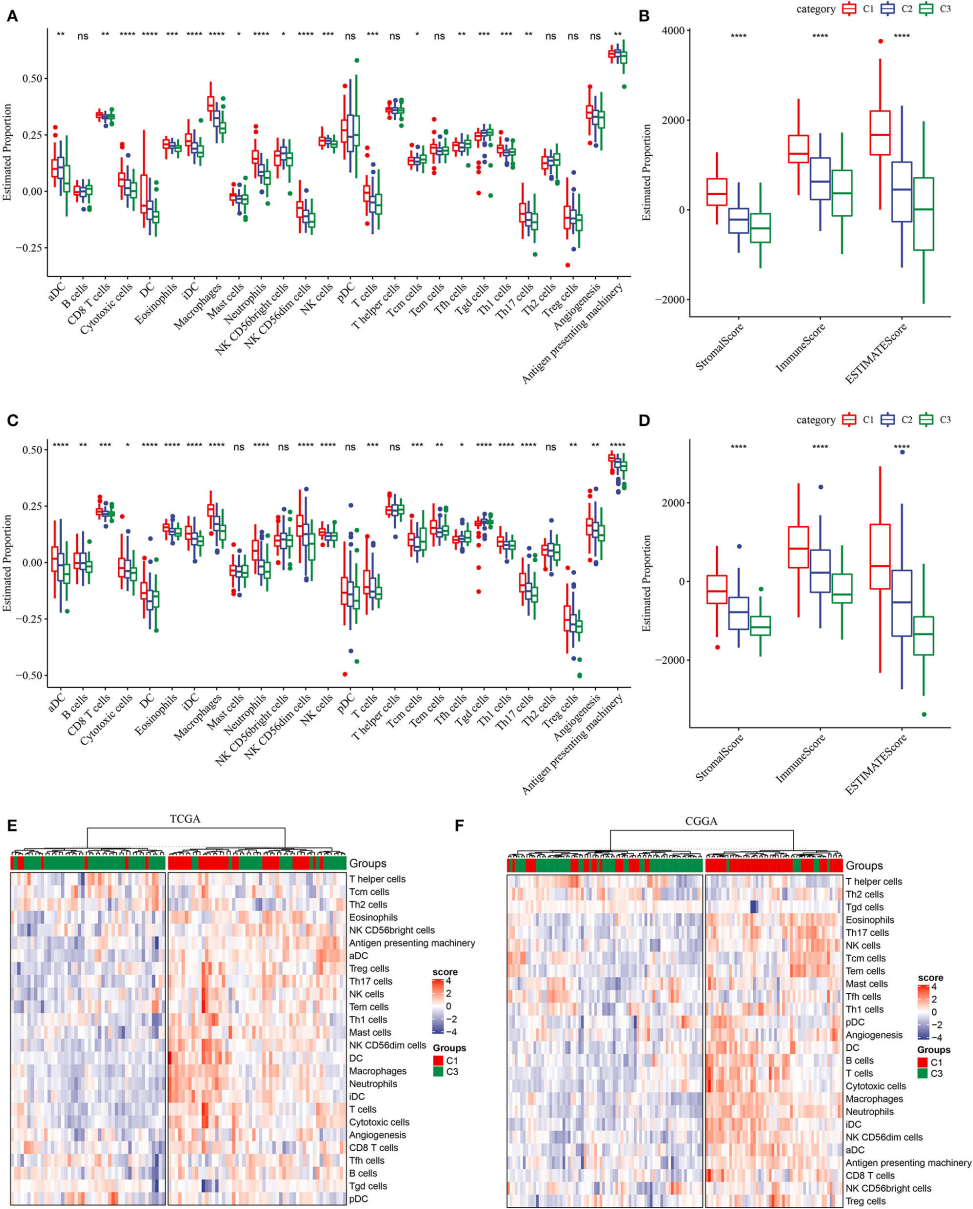
Description of tumor microenvironment (TME) in three subtypes. **(A)** Estimated proportion (ssGSEA score) of immune cells through GSEA in TCGA-GBM dataset. **(B)** Stromal score and immune score of three subtypes calculated by ESTIMATE in TCGA-GBM dataset. **(C)** Estimated proportion (ssGSEA score) of immune cells through GSEA in CGGA-GBM dataset. **(D)** Stromal score and immune score of three subtypes calculated by ESTIMATE in CGGA-GBM dataset. **(E,F)** Unsupervised consensus clustering of immune cells for C1 and C3 subtypes. The Kruskal–Wallis test was conducted. ns, no significance. *p < 0.05, **p < 0.01, ***p < 0.001, ****p < 0.0001.

In most of cancer types, high immune infiltration of lymphocytes indicates favorable survival. However, due to immunosuppressive microenvironment of the brain, the relation between immune infiltration and survival is controversial in GBM, with most references support the conclusion that high immune infiltration is correlated with worse survival (38, 39). To reveal the relation between subtypes and immune infiltration, we obtained gene signatures of 24 immune cell types from previous research (27) and evaluated their expression levels to estimate the proportion of these immune cells. The result showed that C1 subtype had higher proportion of most immune cells, such as dendritic cells (DCs), CD8 T cells, cytotoxic cells, macrophages, natural killer (NK) cells, and regulatory T (Treg) cells (Figures 4A,B). In addition, high activity of angiogenesis and antigen presenting in C1 subtype also facilitated the formation of unfavorable TME. The highest stromal score and immune score were also exhibited in C1 subtype (*p* < 0.0001, Figures 4C,D), supporting the result in previous studies that high immune infiltration was related to unfavorable survival. Compared with normal samples, tumor samples exhibited significantly higher immune infiltration, especially in recurrent samples (*p* < 0.001, Supplementary Figure S3). Additionally, unsupervised consensus clustering on gene signatures of immune cells showed the same conclusion that C1 subtype obviously had higher immune infiltration than C3 subtype (Figures 4E,F). Therefore, we inferred that hypoxia-related lncRNAs may modulate TME through immune-related pathways.

### Distinct Expression of Immune Checkpoints in Three Subtypes

**Figure 5:**
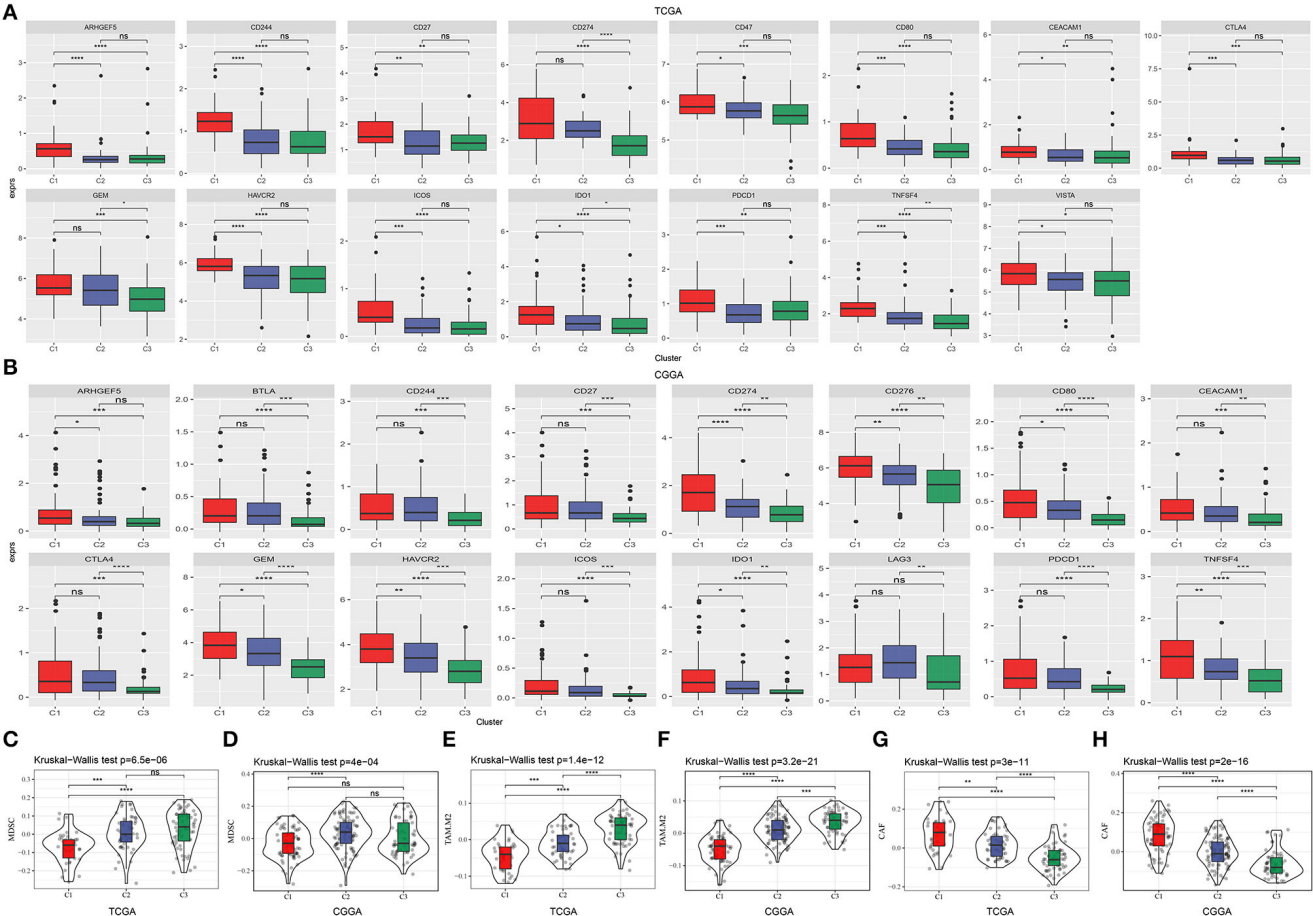
Expression of immune checkpoints and the proportion of critical immune cells in three subtypes. **(A,B)** Immune checkpoints that were differentially expressed among three subtypes in TCGA-GBM and CGGA-GBM datasets. The vertical axis indicates log (TPM+1) expression value. ANOVA was conducted. **(C–H)** Estimated proportion of myeloid-derived suppressor cells (MDSCs), M2 tumor-associated macrophages (TAMs) and cancer-associated fibroblasts (CAFs) in TCGA-GBM and CGGA-GBM datasets. The Kruskal–Wallis test was conducted. ns, no significance. *p < 0.05, **p < 0.01, ***p < 0.001, ****p < 0.0001.

To further understand the mechanism of TME modulation, we assessed the expression of 21 immune checkpoints for all samples (28). Obviously, most of immune checkpoints were highly expressed in C1 subtype (Supplementary Figure S4), indicating activated immune response and active communication between immune-related cells. Among these immune checkpoints, 13 of 21 were all highly expressed in C1 subtype in both two datasets, including ARHGEF5, CD244, CD27, CD274, CD80, CEACAM1, CTLA4, GEM, HAVCR2, ICOS, IDO1, PDCD1, and TNFSF4 (Figures 5A,B). Upregulation of some immune checkpoints, such as CD27, CD274 (PD-L1), CTLA4, IDO1, and PDCD1 (PD-1), were reported to be associated with poor survival and recurrence in gliomas (40–43).

Myeloid-derived suppressor cells and M2 TAMs are believed to contribute to immune suppression in GBM (38). In both two datasets, we observed relatively low enrichment of MDSCs and M2 macrophages in C1 subtype (Figures 5C–F). CAFs are illustrated to be implicated in angiogenesis, tumor growth, migration, and malignancy (44). In our results, CAFs were shown to be the most accumulated in C1 subtype (Figures 5G,H), supporting that CAFs play critical roles in leading to tumor-beneficial TME and tumor progression. Consequently, these results further supported the reasonability of our molecular subtyping and indicated the important role of hypoxia-related lncRNAs in regulating TME.

### Hypoxia-Related LncRNAs Are Involved in Tumorigenesis Through Regulating Transcriptional Factors

**Figure 6:**
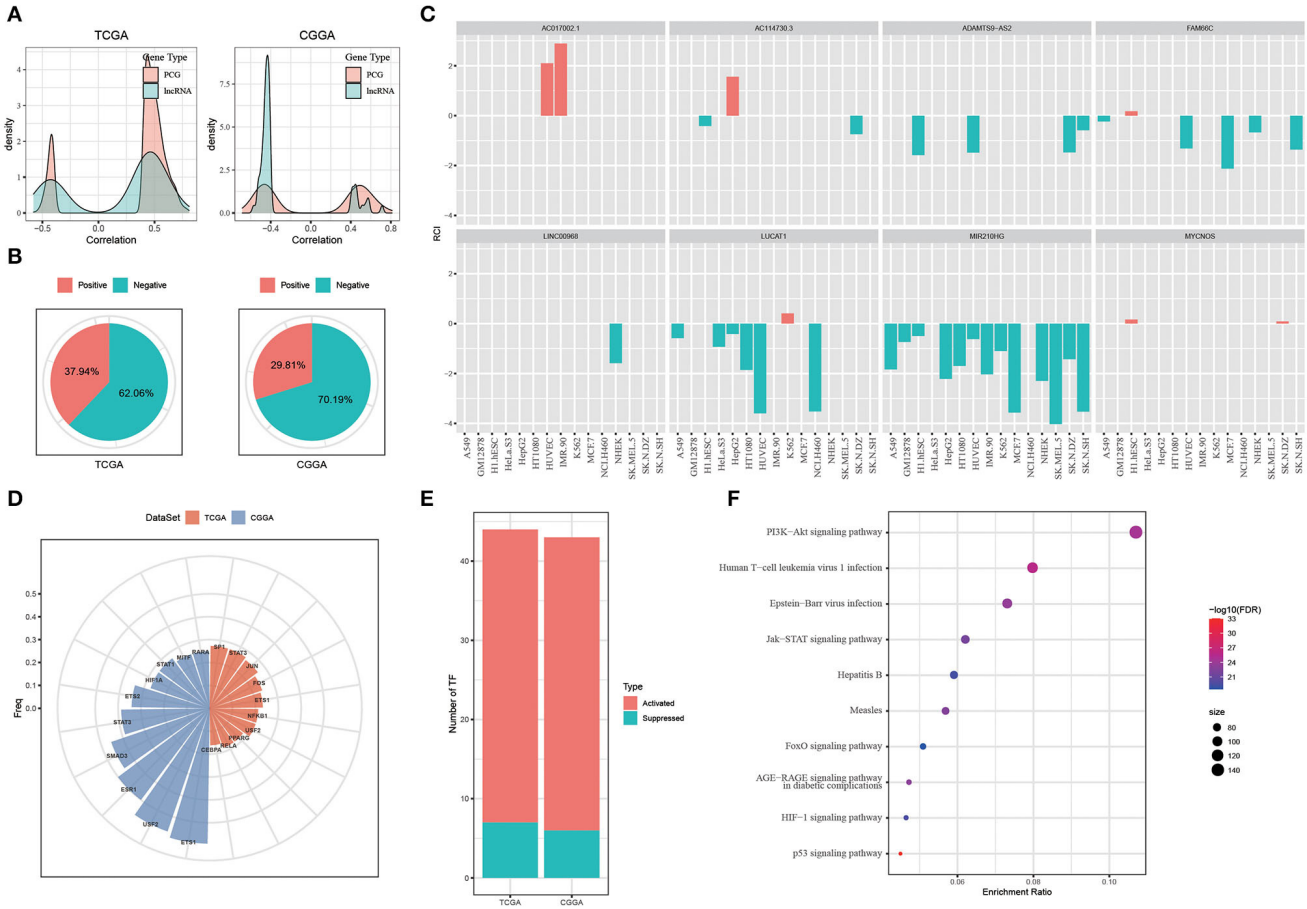
Exploring the function of hypoxia-related long non-coding RNAs (lncRNAs). **(A)** Correlation between hypoxia-related lncRNAs and PCGs. **(B)** Location of hypoxia-related lncRNAs analyzed by LncATLAS. Positive indicates RCI > 0 and negative indicates RCI < 0. **(C)** The location of the 8 identified hypoxia-related lncRNAs. Red indicates RCI > 0 and green indicates RCI < 0. **(D)** The top 10 TFs negatively correlated to nuclear lncRNAs. **(E)** The number of activated and suppressed TFs by comparing C1 with C3. **(F)** The top 10 enriched pathways of upregulated TFs in C1 subtype. Size indicates gene counts.

In the previous sections, we identified eight hypoxia-related lncRNAs and developed three molecular subtypes based on these lncRNAs. Oncogenic pathways and immune-related pathways were highly enriched in C1 subtype (Figure 3). Notably, C1 subtype showed high immune infiltration and high expression of immune checkpoints (Figures 4, 5). Therefore, we suspected that hypoxia-related lncRNAs were involved in promoting tumorigenesis possibly by regulating expression of genes contributing to tumor progression. To verify our hypothesis, we next analyzed the relation between hypoxia-related lncRNAs and protein-coding genes (PCGs). Significant correlations of whether positive or negative were observed between them in both two datasets (Figure 6A), suggesting that hypoxia-related lncRNAs served as regulators on PCG expression.

Long non-coding RNAs regulate gene expression through various ways largely depending on the location of lncRNAs. We then applied LncATLAS to assess the location of hypoxia-related lncRNAs. According to relative concentration index (RCI), we found that most of lncRNAs located in the nuclear (negative, RCI < 0), with 62.06% in TCGA-GBM dataset and 70.19% in CGGA-GBM dataset (Figure 6B). Of eight hypoxia-related lncRNAs using for subtyping, six of them located in the nuclear besides AC017002.1 and MYCNOS only locating in the cytoplasm (Figure 6C). LncRNAs located in the nuclear were reported to regulate gene expression through interacting with genes directly or recruiting TFs (45). Therefore, we next evaluated the TF activity related to the six lncRNAs in three subtypes based on the algorithm developed by Garcia-Alonso et al. (31). As a result, a total of 52 and 48 TFs in TCGA and CGGA datasets, respectively, were screened to be differentially expressed among three subtypes (Supplementary Tables S2, S3). Correlation analysis between differential TFs and nuclear-located lncRNAs identified the top 10 TFs that were all negatively correlated with lncRNA expression (*p* < 0.05, Figure 6D).

**Figure 7:**
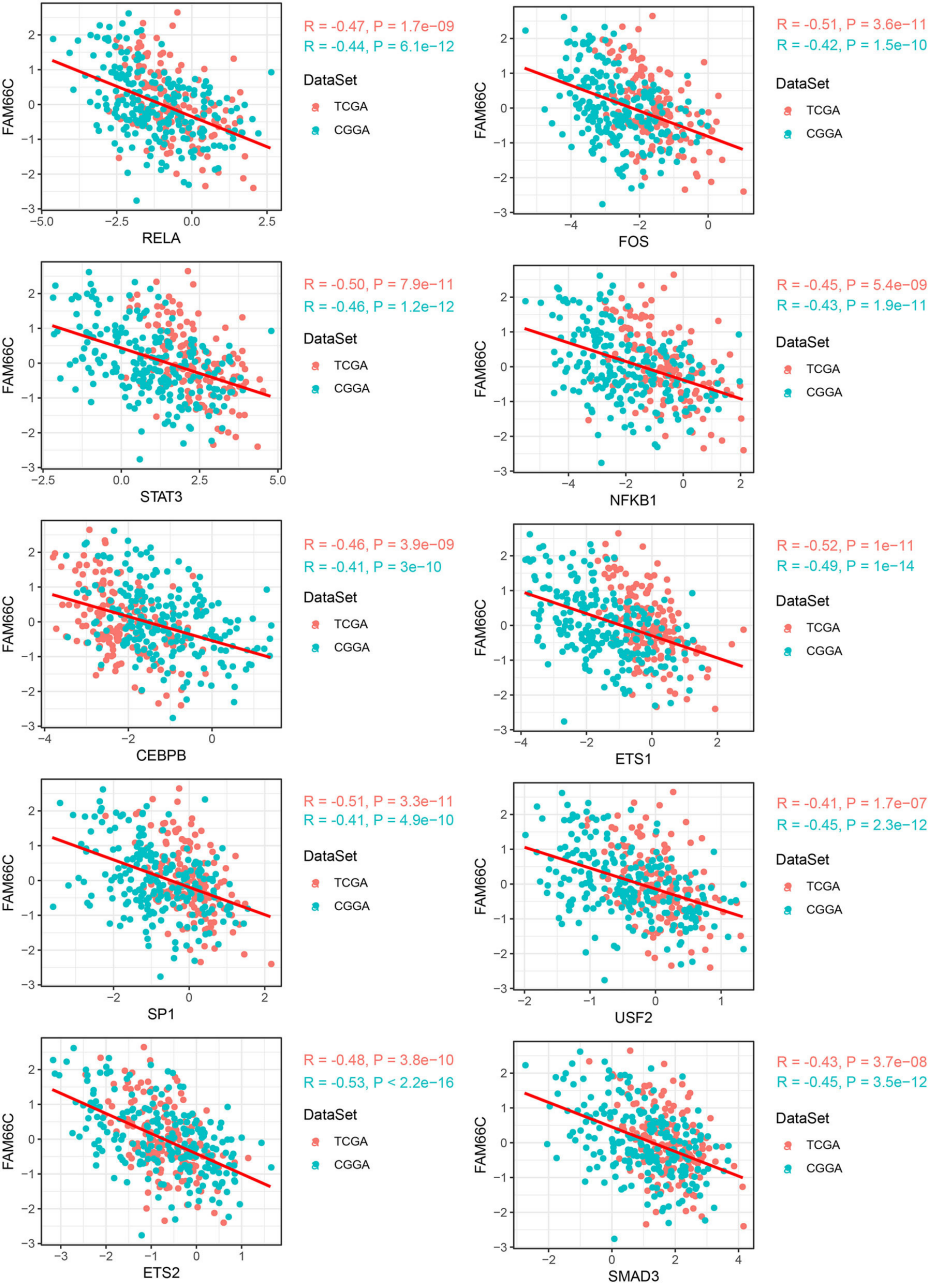
LncRNA–TF pairs that exhibited negative correlation (*R* < −0.3) in both two datasets. Horizontal axis indicates TFs and vertical axis indicates lncRNAs. PCG, protein-coding gene; TF, transcription factor; RCI, relative concentration index.

By comparing the TF activity between C1 and C3 subtypes, we found the majority of TFs were relatively upregulated in C1 subtype (Figure 6E), and 33 TFs were upregulated in both two datasets. We inferred that these 33 TFs played key roles in tumor progression, and thus implemented enrichment analysis on them to identify key pathways. Not surprisingly, the result showed that 33 TFs were highly enriched in oncogenic pathways including PI3K-Akt signaling, JAK-STAT signaling, HIF-1 signaling, and p53 signaling pathways (Figure 6F). Among 33 upregulated TFs, we observed that lncRNA-FAM66C was strongly and negatively correlated with 10 of 33 TFs including RELA, FOS, STAT3, NFKB1, CEBPB, ETS1, SP1, USF2, ETS2, and SMAD3 (*R* < −0.3, *p* < 0.05, Figure 7). Some of these TFs have been abundantly reported to regulate immune response, such as RELA, STAT3 and SMAD3, suggesting that lncRNA-FAM66C plays a pivotal role in orchestrating immune response and TME.

### Identifying Three Key Hypoxia-Related LncRNAs With Prognostic Value

**Figure 8:**
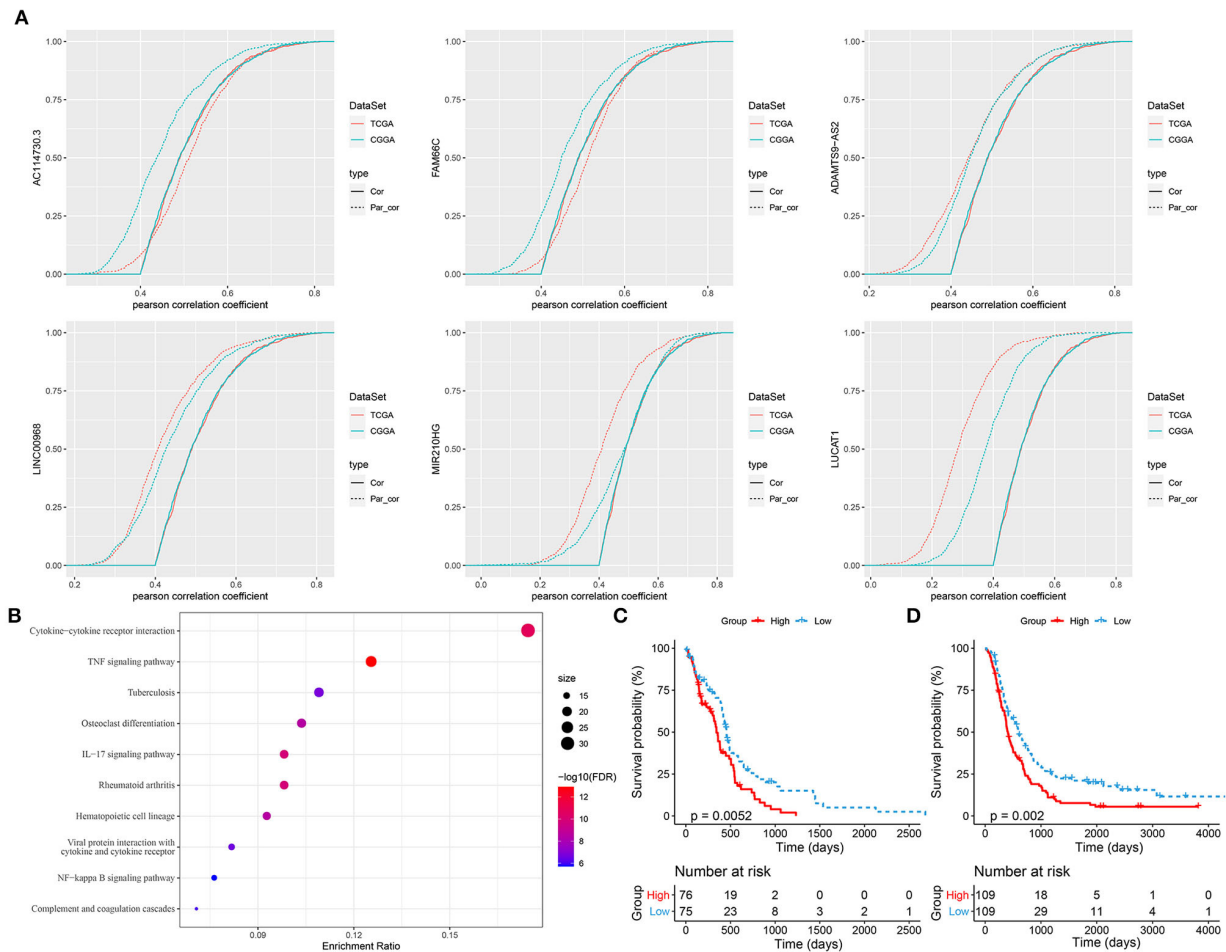
Identifying key hypoxia-related long non-coding RNAs (lncRNAs). **(A)** First-order partial correlation analysis among hypoxia score, expression of hypoxia-related genes, and lncRNAs. Green and red curves indicate GSE108474 and TCGA-GBM datasets, respectively. Solid and dotted curves indicate the correlation between hypoxia score and expression of hypoxia-related genes before and after removing the effect of lncRNAs, respectively. **(B)** GSEA of hypoxia-related genes associated with three key lncRNAs. Size indicates gene counts. **(C,D)** Kaplan–Meier survival plots of high-risk and low-risk groups classified by three key lncRNAs in TCGA-GBM **(C)** and CGGA-GBM **(D)** datasets.

As hypoxia-related lncRNAs were illustrated to be highly associated with hypoxia score, oncogenic pathways and prognosis, we attempted to identify key lncRNAs serving as prognostic indicators based on first-order partial correlation analysis. According to the correlation analysis among hypoxia score, the expression of hypoxia-related genes, and the six lncRNAs, we identified three key lncRNAs (ADAMTS9-AS2, LINC00968, and LUCAT1) that significantly affected the correlation between hypoxia score and the expression of hypoxia-related genes when eliminating three lncRNAs in both TCGA-GBM and CCGA-GBM datasets (Figure 8A). GSEA on hypoxia-related genes with relation to all three lncRNAs revealed that immune-related pathways were significantly enriched including cytokine–cytokine receptor interaction, IL-17 signaling pathway, TNF signaling pathway, NF-kappa B signaling pathway, and complement and coagulation cascades (Figure 8B). In the relation between immune cells and the three lncRNAs, we observed that the expression of three lncRNAs were all significantly associated with the enrichment of macrophages (*p* < 0.05, Supplementary Figure S5). In addition, LINC00968 and LUCAT1 were also significantly correlated with neutrophils and immature DCs (iDCs), whereas cytotoxic cells and DCs were only correlated with LUCAT1 (*p* < 0.01, Supplementary Figure S5). In a pan-cancer analysis of the relation between LINC00968 and immune infiltration, we found its expression also strikingly correlated with the immune infiltration in other cancer types (Supplementary Table S4). Moreover, we established a prognostic model based on the three lncRNAs (Risk Score = 0.1782968^*^ADAMTS9-AS2 + 0.1510304^*^LINC00968 + 0.1551735^*^LUCAT1), and found that these three lncRNAs were effective to classify samples into high-risk and low-risk groups in both TCGA-GBM and CGGA-GBM datasets (Figures 8C,D, *p* = 0.0052 and *p* = 0.002, respectively). In addition, receiver operating characteristic (ROC) analysis showed that the model had a favorable performance in predicting prognosis for GBM patients (Supplementary Figure S6).

## Discussion

Hypoxia is involved in the formation of cancer hallmarks such as angiogenesis, TME modulation, and dysregulated metabolism (10). In the present study, we underlined the critical role of hypoxia for GBM development through describing the associations among hypoxia-related genes, hypoxia-related lncRNAs, TME, and oncogenic pathways. Importantly, we expanded the possible mechanism of hypoxia in contributing to modulate TME and promote tumorigenesis.

Based on the eight identified hypoxia-related lncRNAs, we constructed three molecular subtypes with differential OS. IDH mutations were found to be the most in C3 subtype, supporting the previous finding that mutant IDH was a favorable factor for OS and progression-free survival in GBM patients (46). It has been reported that IDH-wt glioma stem cells (GSCs) presented larger transcriptomic changes than IDH-mutant GSCs in response to hypoxia (47). This significant difference indicates different mechanisms of two IDH status to hypoxia, which may contribute to different extent of tumor cell growth.

In the comparison of enriched pathways between C1 and C3 subtypes, a number of oncogenic pathways were significantly upregulated in C1 subtype, such as EMT, TNF-α, hypoxia, angiogenesis, and glycolysis. It was reasonable that hypoxia pathway was more activated in C1 subtype, which proved that the 8 hypoxia-related lncRNAs contributed an essential part in regulating hypoxia-related genes. Hypoxia activates HIF-1 whose overexpression is strongly associated with facilitated tumor cell migration, metastasis, and angiogenesis (48). EMT is one of basic mechanisms for tumor cell migration, and HIF-1 pathway is considered as the most important one for hypoxia-induced EMT (49). In the HIF-1 signaling, immune modulators, such as interleukin-1 (IL-1) and TNF-α, serve as stimulators to promote HIF-1 expression (50). Furthermore, HIF-1 is involved in regulating many genes responsible for glucose metabolism, which promotes glycolysis through driving phase transition of key glycolytic enzymes (51). These cascade responses induced by hypoxia result in the invasive tumor cells and progressive phenotypes corresponding to C1 subtype.

Tumor microenvironment is known as the important aspect for understanding anti-tumor response and sensitivity to immunotherapy. In solid tumors, the existence of hypoxia is also followed by the TME modulation. Highly enriched immune-related pathways in C1 subtype such as interferon gamma response, inflammatory response, IL6-JAK-STAT3, and IL2-STAT5 suggested a close relation between hypoxia and these pathways. Actually, C1 subtype also manifested significant upregulation of a number of immune checkpoints, especially CD27, CD274 (PD-L1), CTLA4, IDO1, and PDCD1 (PD-1) whose overexpression was associated with immunosuppressive response. PD-L1 was demonstrated to be upregulated under HIF-1 promotion that activated by hypoxia (52). Heiland et al. found that activated JAK/STAT signaling and CD274 expression were both shown in tumor-associated astrocytes (40). The inhibition of JAK/STAT pathway restored activated microenvironment from the immunosuppressive microenvironment and reduced tumor cell proliferation in tumor-associated astrocytes. Therefore, we guessed that HIF-1 upregulated PD-L1 expression through activating JAK-STAT signaling. In this process, hypoxia-related lncRNAs may serve as important roles in modulating gene expression.

Among the eight lncRNAs, some of them have been reported to be involved in cancer migration and progression in many cancer types. For example, ADAMTS9-AS2 is considered as a tumor suppressor in inhibiting the migration of glioma cells, with the regulation by DNMT1 (53). ADAMTS9-AS2 upregulation also suppresses cancer cell progression in lung cancer (54), liver cancer (55), and gastric cancer (56). LINC00968 serves as oncogenic or tumor-suppressive roles in different cancer types. In breast cancer, LINC00968 can attenuate cancer cell proliferation, migration, and angiogenesis (57). However, LINC00968 promotes the progression of epithelial ovarian cancer regulating by ERK and AKT pathways (58). The lung cancer-related transcript 1 (LUCAT1) has been numerously reported in contributing cancer cell proliferation, migration, and invasion in breast cancer, liver cancer, ovarian cancer, and so on, which is recognized as a potential prognostic biomarker (59). MIR210HG is also identified as a prognostic lncRNA in glioma (60), hepatocellular carcinoma (61), and colorectal adenocarcinoma (62). FAM66C is not much reported compared to the above lncRNAs, but it is illustrated to promote cell proliferation by suppressing proteasome pathway in prostate cancer, promote cancer progression in cholangiocarcinoma and pancreatic cancer (63–65). MYCNOS also functions as an oncogenic role in promoting GBM cell proliferation and hepatocellular carcinoma invasion (66, 67).

As our molecular subtypes based on the eight hypoxia-related lncRNAs exhibited consistent results with previous research, we tried to further parse the role of hypoxia-related lncRNAs in GBM development. As a consequence, we identified that 33 TFs were significantly upregulated in C1 subtype comparing with C3 subtype. Not surprisingly, JAK-STAT signaling and HIF-1 signaling pathways were significantly enriched in these upregulated TFs, indicating the key role of hypoxia-related lncRNAs in TME modulation possibly by regulating the expression of 33 TFs. Notably, we identified 10 TFs that were strongly correlated with lncRNA-FAM66C (one of the eight hypoxia-related lncRNAs). Among these TFs, RELA was demonstrated to upregulate HIF-1α expression and EMT in GBM (68). FOS was identified as a hypoxia-induced gene in a malignant glioma cell line (69). CEBPB (70), ETS-1 (71), SP1 (72), USF2 (73), and SMAD3 (74) were all reported to be associated with hypoxia response in GBM or other solid tumors. Particularly, STAT3 was also identified to be highly associated with lncRNA-FAM66C. The results strongly supported that lncRNA-FAM66C was a pivotal regulator contributing to the network in hypoxia-related pathways. So far, no research has reported the function of FAM66C in GBM development. To further demonstrate its role in hypoxia and tumorigenesis, strengthened experiments are needed in future.

In addition, we screened three key lncRNAs with prognostic value for predicting GBM OS in clinical. These three lncRNAs (ADAMTS9-AS2, LINC00968, and LUCAT1) were also shown to be key regulators of hypoxia-related genes that were involved in TNF signaling, IL-17 signaling, and NF-kappa B signaling pathways.

In conclusion, this study parsed the role of hypoxia-related lncRNAs in hypoxia, TME and tumorigenesis based on comprehensive analysis of three molecular subtypes for GBM. Importantly, we explored the possible mechanisms of hypoxia-related lncRNAs in modulating oncogenic pathways and immune-related pathways. Within the eight hypoxia-related lncRNAs, FAM66C was identified as a critical regulator in hypoxia-related pathways. Finally, we constructed a prognostic model according to three key lncRNAs that could act as predictive biomarkers for GBM patients. However, the non-negligible limitation of our study was that only pure bioinformatics analysis was performed. In the future study, wet experiments in more clinical samples were needed to be figured out for demonstrating our results.

## Data Availability Statement

The datasets presented in this study can be found in online repositories. The names of the repository/repositories and accession number(s) can be found in the article/Supplementary Material.

## Author Contributions

YY designed the study and reviewed and edited the manuscript. NQ and QF contributed to the literature research. CL and LZ analyzed and interpreted the data. DL and YW wrote the initial draft of the manuscript. All authors read and approved the manuscript.

## Conflict of Interest

The authors declare that the research was conducted in the absence of any commercial or financial relationships that could be construed as a potential conflict of interest. The reviewer XL declared a shared affiliation with the author QF to the handling editor at the time of review.

## Publisher's Note

All claims expressed in this article are solely those of the authors and do not necessarily represent those of their affiliated organizations, or those of the publisher, the editors and the reviewers. Any product that may be evaluated in this article, or claim that may be made by its manufacturer, is not guaranteed or endorsed by the publisher.
