# Supplementary material for: LncRNA-FAM66C Was Identified as a Key Regulator for Modulating Tumor Microenvironment and Hypoxia-Related Pathways in Glioblastoma
Source: Front Public Health. 2022 Jul 6;10:898270. doi: 10.3389/fpubh.2022.898270 (PMC9299378; doi:10.3389/fpubh.2022.898270)
Supplement: Supplementary file 14 [file Data_Sheet_1.PDF]

Supplementary materials

Supplementary Figure S1. The flow chart of this study.

Supplementary Figure S2. Genomic features of three subtypes. (A) The score of aneuploidy, homologous recombination defects, fraction altered, number of segments, and tumor mutation burden. (B) Pearson correlation analysis between different genomic features and hypoxia score. (C) Mutation frequencies of the top 20 significantly altered genes in three subtypes. Mut, mutants; Del, deletions; Amp, amplifications. ns, no significance. *$P < 0.05$, **$P < 0.01$.

Supplementary Figure S3. ESTIMATE analysis of normal, primary, and recurrent samples in TCGA-GBM dataset. Kruskal-Wallis test was conducted. ***$P < 0.001$.

Supplementary Figure S4. Expression of 21 immune checkpoints of three subtypes in two datasets.

Supplementary Figure S5. Supplementary Figure S5. Pearson correlation analysis between immune cells and three key lncRNAs in TCGA-GBM dataset. *$P < 0.05$, **$P < 0.01$, ***$P < 0.001$.

Supplementary Figure S6. ROC curve of the three-lncRNA prognostic model in TCGA-GBM (A) and CGGA-GBM (B) datasets.

Supplementary Table S1. Correlation coefficients of 8 identified hypoxia-related lncRNAs in two datasets.

Supplementary Table S2. 52 differentially expressed TFs among three subtypes in TCGA-GBM dataset.

Supplementary Table S3. 48 differentially expressed TFs among three subtypes in CGGA-GBM dataset.

Supplementary Table S4. Correlation analysis between LINC00968 and immune infiltration (ESTIMATE analysis) in 39 TCGA cancer types.

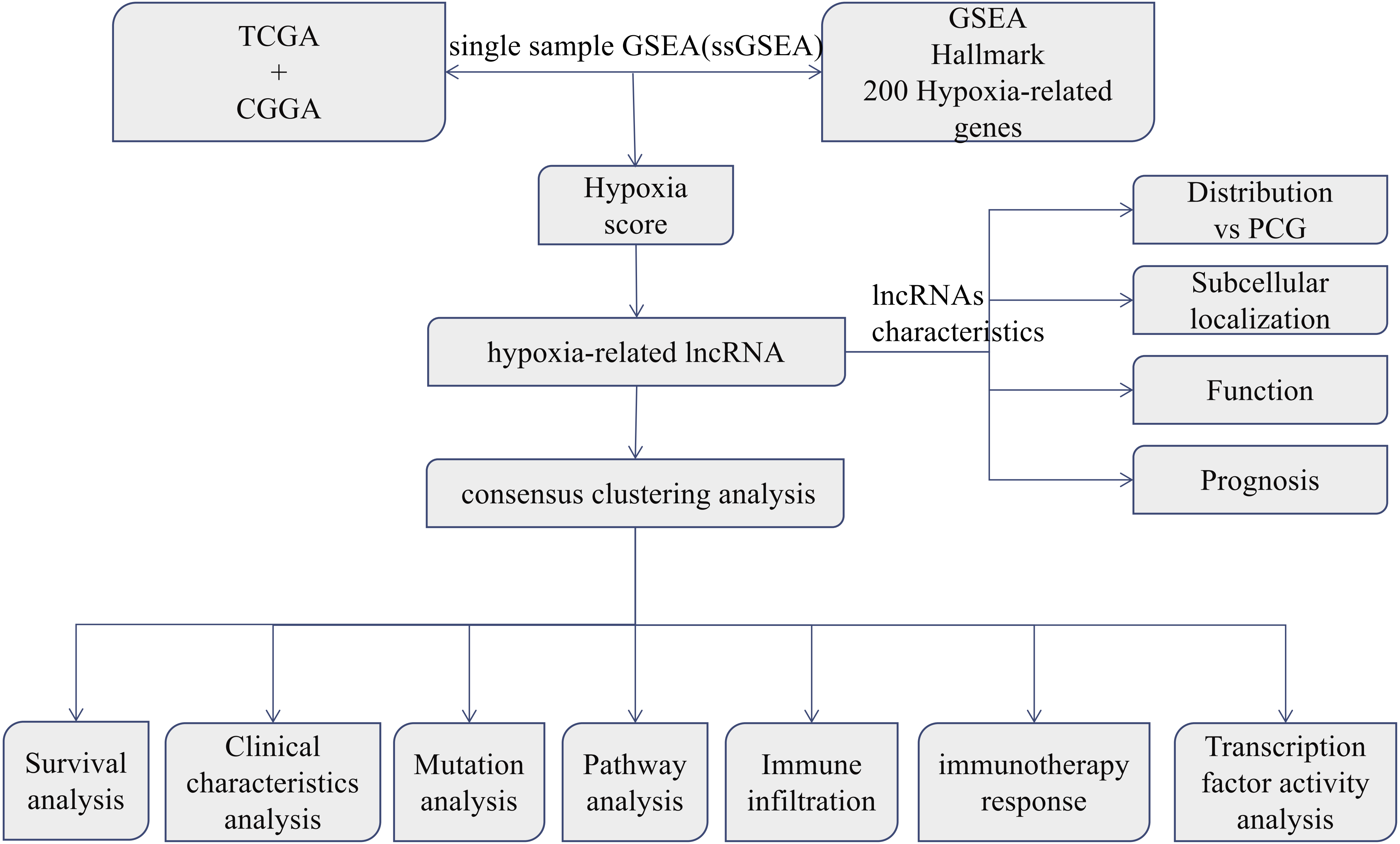
TCGA
+
CGGA
single sample GSEA(ssGSEA)
GSEA
Hallmark
200 Hypoxia-related genes
Hypoxia score
hypoxia-related lncRNA
lncRNAs characteristics
Distribution vs PCG
Subcellular localization
Function
Prognosis
consensus clustering analysis
Survival analysis
Clinical characteristics analysis
Mutation analysis
Pathway analysis
Immune infiltration
immunotherapy response
Transcription factor activity analysis

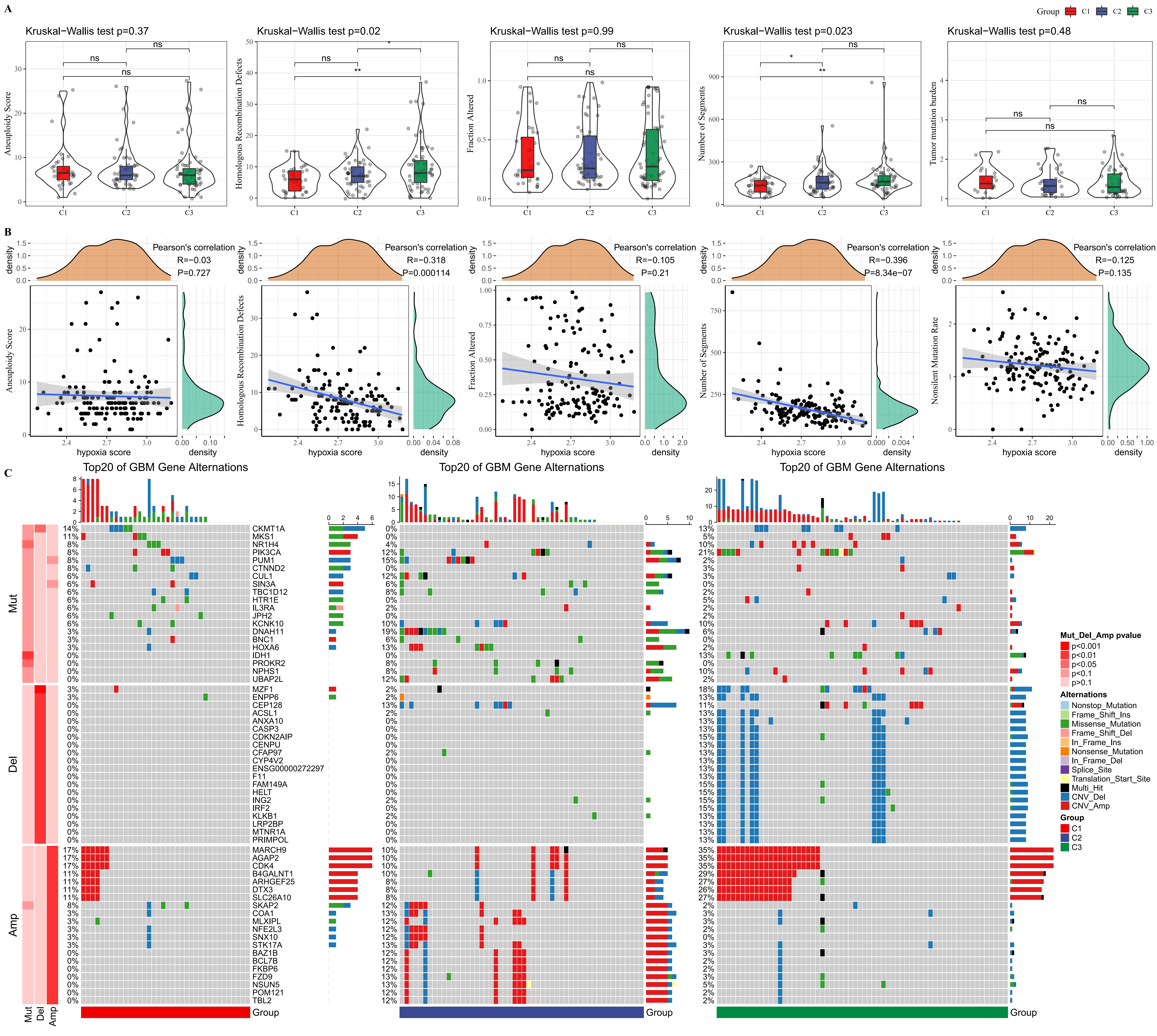
A
Group C1 C2 C3
Kruskal−Wallis test p=0.37
Aneuploidy Score
Kruskal−Wallis test p=0.02
Homologous Recombination Defects
Kruskal−Wallis test p=0.99
Fraction Altered
Kruskal−Wallis test p=0.023
Number of Segments
Kruskal−Wallis test p=0.48
Tumor mutation burden
ns
*
**
C1
C2
C3
B
Pearson's correlation
R=−0.03
P=0.727
R=−0.318
P=0.000114
R=−0.105
P=0.21
R=−0.396
P=8.34e−07
R=−0.125
P=0.135
density
hypoxia score
Aneuploidy Score
Homologous Recombination Defects
Fraction Altered
Number of Segments
Nonsilent Mutation Rate
C
Top20 of GBM Gene Alternations
Mut
Del
Amp
CKMT1A
MKS1
NR1H4
PIK3CA
PUM1
CTNND2
CUL1
SIN3A
TBC1D12
HTR1E
IL3RA
JPH2
KCNK10
DNAH11
BNC1
HOXA6
IDH1
PROKR2
NPHS1
UBAP2L
MZF1
ENPP6
CEP128
ACSL1
ANXA10
CASP3
CDKN2AIP
CENPU
CFAP97
CYP4V2
ENSG00000272297
F11
FAM149A
HELT
ING2
IRF2
KLKB1
LRP2BP
MTNR1A
PRIMPOL
MARCH9
AGAP2
CDK4
B4GALNT1
ARHGEF25
DTX3
SLC26A10
SKAP2
COA1
MLXIPL
NFE2L3
SNX10
STK17A
BAZ1B
BCL7B
FKBP6
FZD9
NSUN5
POM121
TBL2
Group
Mut_Del_Amp pvalue
p<0.001
p<0.01
p<0.05
p<0.1
p>0.1
Alternations
Nonstop_Mutation
Frame_Shift_Ins
Missense_Mutation
Frame_Shift_Del
In_Frame_Ins
Nonsense_Mutation
In_Frame_Del
Splice_Site
Translation_Start_Site
Multi_Hit
CNV_Del
CNV_Amp
Group
C1
C2
C3

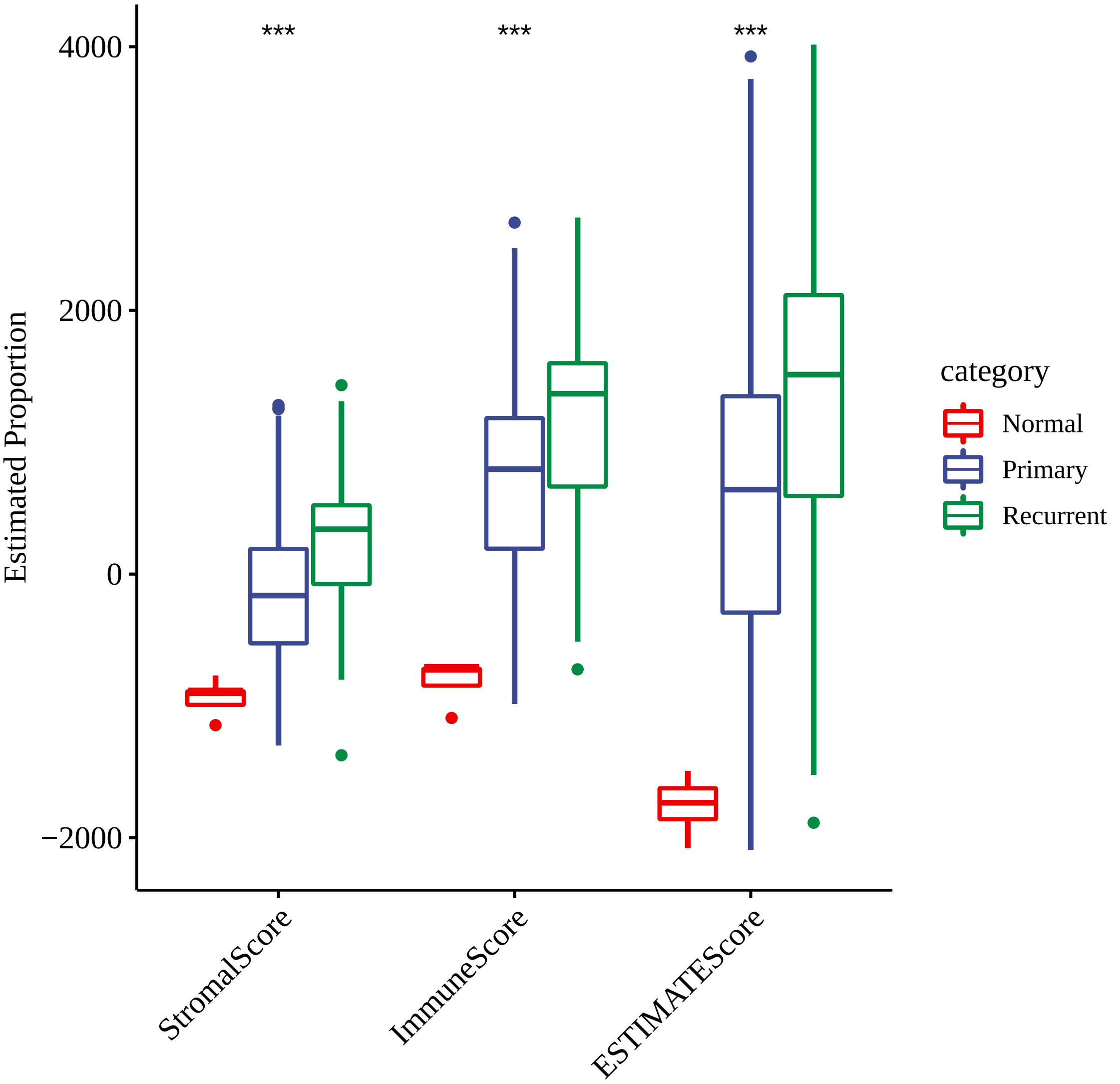
***
***
***
4000
2000
0
−2000
Estimated Proportion
StromalScore
ImmuneScore
ESTIMATEScore
category
Normal
Primary
Recurrent

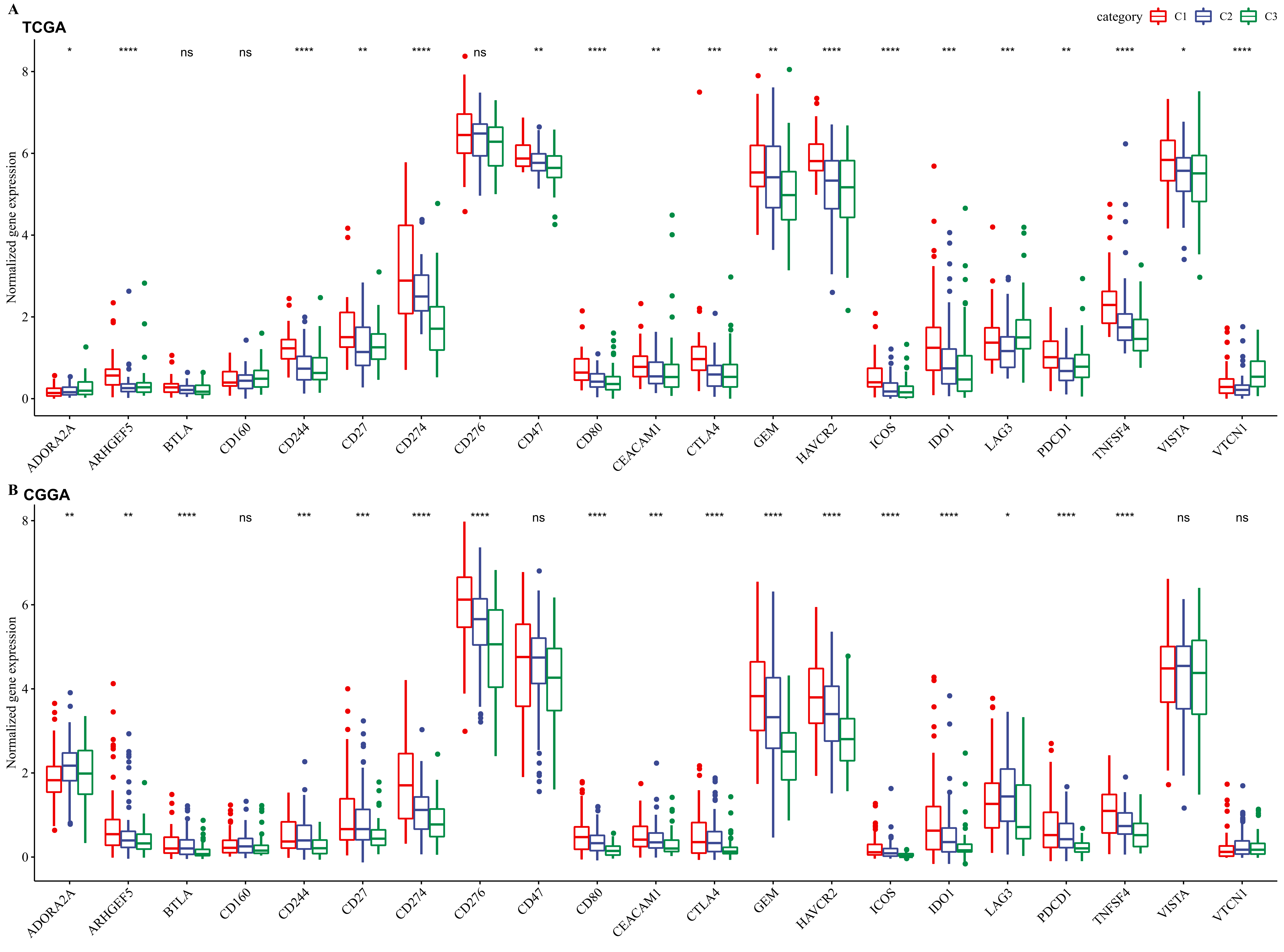
A
TCGA
category C1 C2 C3
Normalized gene expression
0
2
4
6
8
* **** ns ns **** ** **** ns ** **** ** *** ** **** **** *** *** ** **** * ****
ADORA2A
ARHGEF5
BTLA
CD160
CD244
CD27
CD274
CD276
CD47
CD80
CEACAM1
CTLA4
GEM
HAVCR2
ICOS
IDO1
LAG3
PDCD1
TNFSF4
VISTA
VTCN1
B
CGGA
Normalized gene expression
0
2
4
6
8
** ** **** ns *** *** **** **** ns **** *** **** **** **** **** **** * **** **** ns ns
ADORA2A
ARHGEF5
BTLA
CD160
CD244
CD27
CD274
CD276
CD47
CD80
CEACAM1
CTLA4
GEM
HAVCR2
ICOS
IDO1
LAG3
PDCD1
TNFSF4
VISTA
VTCN1

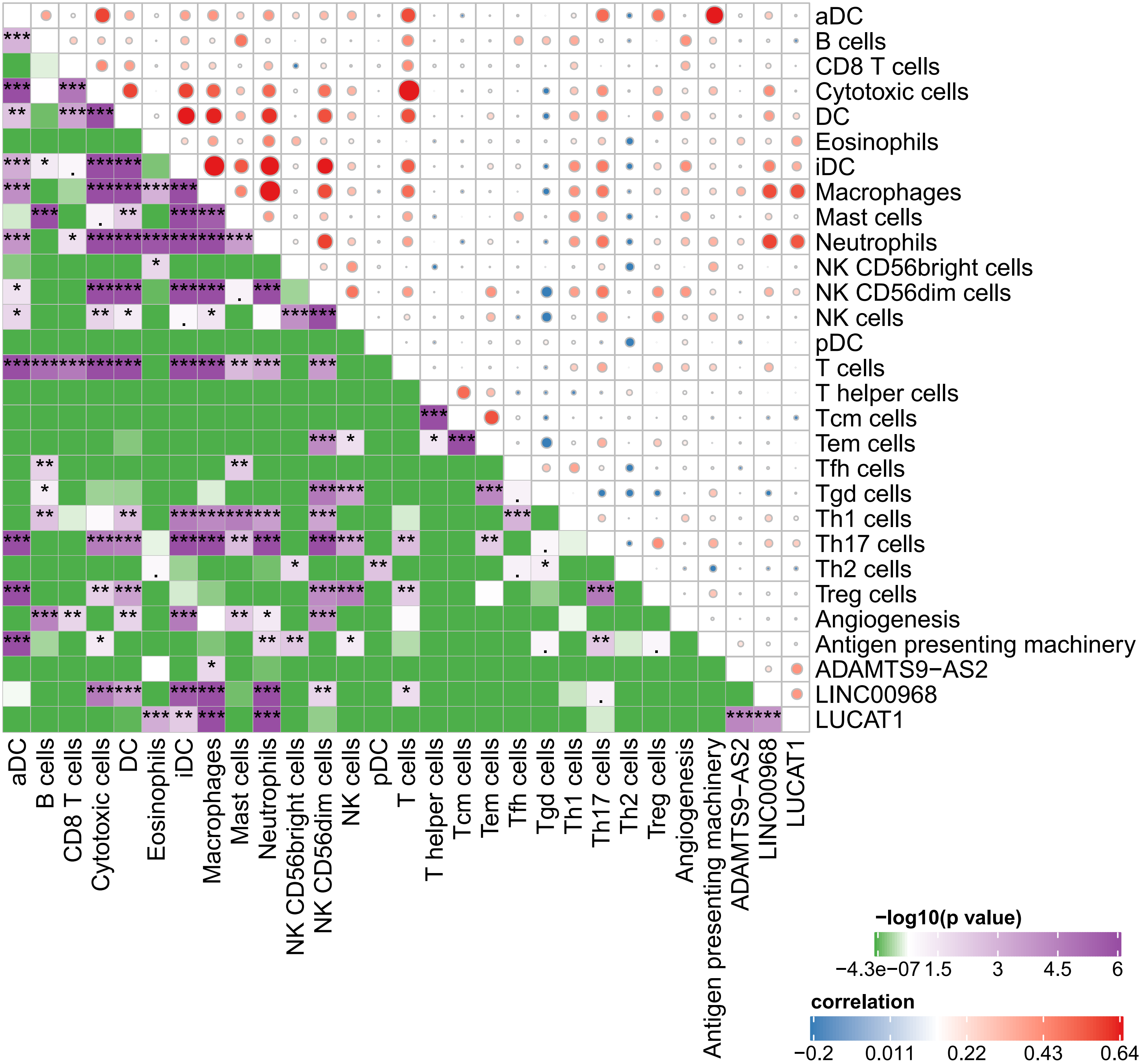
aDC
B cells
CD8 T cells
Cytotoxic cells
DC
Eosinophils
iDC
Macrophages
Mast cells
Neutrophils
NK CD56bright cells
NK CD56dim cells
NK cells
pDC
T cells
T helper cells
Tcm cells
Tem cells
Tfh cells
Tgd cells
Th1 cells
Th17 cells
Th2 cells
Treg cells
Angiogenesis
Antigen presenting machinery
ADAMTS9−AS2
LINC00968
LUCAT1
−log10(p value)
−4.3e−07 1.5 3 4.5 6
correlation
−0.2 0.011 0.22 0.43 0.64

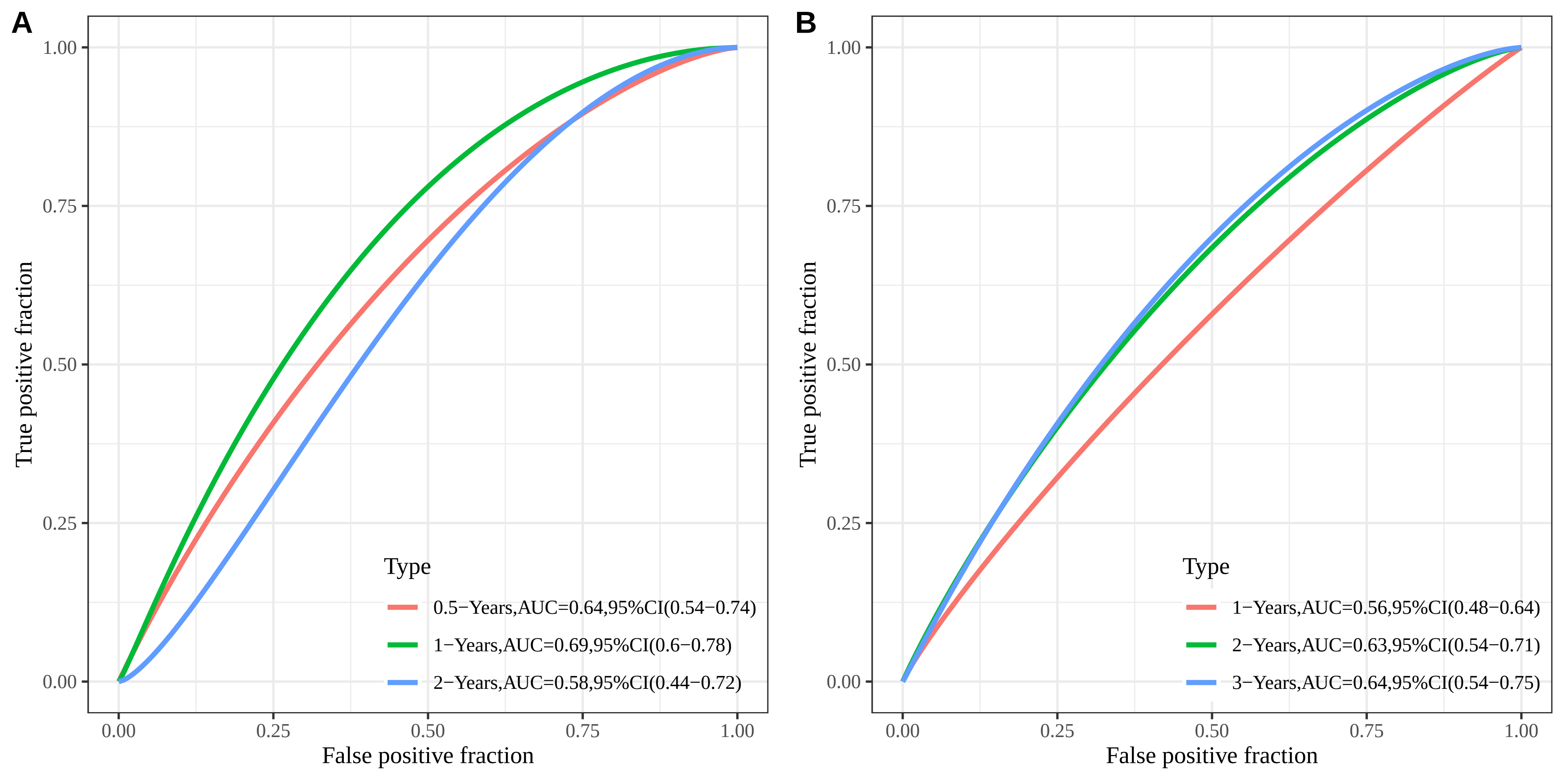
A
B
True positive fraction
False positive fraction
0.00
0.25
0.50
0.75
1.00
Type
0.5−Years,AUC=0.64,95%CI(0.54−0.74)
1−Years,AUC=0.69,95%CI(0.6−0.78)
2−Years,AUC=0.58,95%CI(0.44−0.72)
1−Years,AUC=0.56,95%CI(0.48−0.64)
2−Years,AUC=0.63,95%CI(0.54−0.71)
3−Years,AUC=0.64,95%CI(0.54−0.75)

Supplementary Table S1. Correlation coefficients of 8 identified hypoxia-related lncRNAs in two datasets.

LncRNAs	TCGA	CGGA
AC017002.1	0.436382	0.571725
ADAMTS9-AS2	0.422333	0.448009
LINC00968	0.421637	0.429146
LUCAT1	0.643508	0.712467
MIR210HG	0.586552	0.466055
AC114730.3	-0.42427	-0.42893
FAM66C	-0.47129	-0.4487
MYCNOS	-0.41139	-0.51744

Supplementary Table S2. 52 differentially expressed TFs among three subtypes in TCGA-GBM dataset.

TFs	F.value	p.value	effect.size
AR	3.12	0.0471	0.040461
BACH1	0.626	0.536	0.008385
CEBPA	54.3	2.01E-18	0.423425
CEBPB	53.4	3.39E-18	0.419349
CREB1	15.6	6.99E-07	0.17431
CTCF	10.6	4.92E-05	0.125461
E2F1	23.7	1.16E-09	0.242753
E2F4	21.8	4.96E-09	0.227716
EGR1	4.6	0.0116	0.058474
ELK1	20.8	1.06E-08	0.219712
ESR1	31.8	3.29E-12	0.30045
ETS1	56.8	4.98E-19	0.434193
ETS2	42.4	2.82E-15	0.364104
FOS	87.1	9.96E-26	0.540652
FOXA1	0.17	0.844	0.002286
FOXM1	5.79	0.0038	0.072532
FOXO1	7.8	0.000603	0.095338
FOXO3	18.5	6.65E-08	0.200148
FOXP1	5.23	0.0064	0.065976
GATA2	6.7	0.00163	0.083058
GATA3	0.275	0.76	0.003707
HIF1A	61.9	2.84E-20	0.455671
HNF1A	0.133	0.876	0.001792
HNF4A	17.9	1.09E-07	0.194802
IRF1	14	2.63E-06	0.159412
JUN	76.2	1.76E-23	0.50737
MITF	5.84	0.00362	0.073151
MYB	6.18	0.00263	0.077132
MYC	13.5	4.16E-06	0.154154
NFIC	0.177	0.838	0.002381
NFKB1	77.7	8.48E-24	0.512222
POU2F1	10.9	3.87E-05	0.128273
PPARA	0.64	0.529	0.008571
PPARG	28	4.71E-11	0.274816
PRDM14	21.9	4.53E-09	0.228666
RARA	36.6	1.24E-13	0.330751

RELA	78.8	4.95E-24	0.515754
SMAD3	29.7	1.47E-11	0.286126
SMAD4	27.1	9.69E-11	0.267717
SP1	52.7	5.36E-18	0.41574
SP3	18	9.68E-08	0.196072
SPI1	43.9	1.05E-15	0.372514
STAT1	17.3	1.80E-07	0.189309
STAT2	3.63	0.0288	0.046809
STAT3	71.3	2.06E-22	0.490748
TFAP2A	8.11	0.000455	0.098764
TP53	29.4	1.73E-11	0.284589
USF1	10.6	4.91E-05	0.125477
USF2	53.1	4.24E-18	0.417588
WT1	24.1	8.93E-10	0.245405
YY1	18.4	7.51E-08	0.198828
ZNF263	15.1	1.06E-06	0.16963

Supplementary Table S3. 48 differentially expressed TFs among three subtypes in CGGA-GBM dataset.

TFs	F.value	p.value	effect.size
AR	7.19	0.000952	0.06267
BACH1	7.87	0.000505	0.068176
CEBPA	40.7	9.98E-16	0.274801
CEBPB	27.9	1.66E-11	0.206199
CREB1	4.77	0.00941	0.042476
CTCF	10.9	3.14E-05	0.09194
E2F1	8.14	0.00039	0.070422
E2F4	5.84	0.00339	0.051521
EGR1	16.4	2.35E-07	0.132379
ELK1	27.7	1.95E-11	0.204985
ESR1	52	3.82E-19	0.325983
ETS1	88.4	9.81E-29	0.451147
ETS2	60.5	1.45E-21	0.360054
FOS	61.1	9.66E-22	0.362454
FOXM1	4.55	0.0116	0.040606
FOXO1	1.08	0.341	0.009965
FOXO3	16.5	2.24E-07	0.132745
FOXP1	6.77	0.0014	0.059276
GATA2	3.63	0.0283	0.03263
GATA3	4	0.0196	0.035905
HIF1A	66.9	2.55E-23	0.383654
HNF4A	1.09	0.337	0.010057
IRF1	27.1	3.16E-11	0.201408
JUN	55.9	2.78E-20	0.342218
MITF	16.2	2.86E-07	0.130773
MYC	9.65	9.72E-05	0.082351
NFIC	10.2	6.06E-05	0.086376
NFKB1	61.7	6.49E-22	0.364813
POU2F1	24.6	2.39E-10	0.186249
PPARA	3.42	0.0344	0.030857
PPARG	17.5	8.98E-08	0.140099
PRDM14	22.9	9.90E-10	0.175407
RARA	51.6	4.89E-19	0.324436
RELA	57.5	1.03E-20	0.348291
SMAD3	76.2	9.67E-26	0.414797
SP1	48.1	5.31E-18	0.309282

SP3	51.6	5.02E-19	0.324269
SPI1	34.3	1.17E-13	0.241926
STAT1	56.9	1.47E-20	0.346105
STAT2	13.8	2.40E-06	0.113415
STAT3	76.7	7.34E-26	0.416298
TFAP2A	7.11	0.00102	0.062076
TP53	37.3	1.20E-14	0.257838
USF1	42.8	2.24E-16	0.284811
USF2	63.7	1.84E-22	0.372197
WT1	13.2	4.00E-06	0.109196
YY1	9.86	8.00E-05	0.084018
ZNF263	26.4	5.68E-11	0.197039

Datasets	StromalScore spearman_P	StromalScore spearman_R	ImmuneScore spearman_P	ImmuneScore spearman_R	ESTIMATEScore spearman_P	ESTIMATEScore spearman_R
TCGA-GBM(N=152)	0.610722329	6.59E-17	0.645462872	2.79E-19	0.663874334	1.15E-20
TCGA-GBMLGG(N=656)	0.610027696	4.07E-68	0.616891141	4.87E-70	0.627303764	4.77E-73
TCGA-LGG(N=504)	0.502978874	1.13E-33	0.50549001	4.79E-34	0.516132599	1.17E-35
TCGA-CESC(N=291)	0.496336819	1.67E-19	0.210226241	0.000304791	0.373933951	4.34E-11
TCGA-LUAD(N=500)	0.476769692	9.67E-30	0.374321811	4.47E-18	0.455812269	5.08E-27
TCGA-COAD(N=282)	0.738691593	6.93E-50	0.580821898	7.77E-27	0.704545715	1.33E-43
TCGA-COADREAD(N=373	0.731012886	1.49E-63	0.575877208	2.48E-34	0.696887223	1.62E-55
TCGA-LAML(N=149)	0.600106307	6.06E-16	0.624997055	1.61E-17	0.650234289	2.87E-19
TCGA-BRCA(N=1077)	0.509264299	3.94E-72	0.326100166	4.21E-28	0.450977262	4.50E-55
TCGA-ESCA(N=181)	0.626528355	3.97E-21	0.366322601	3.94E-07	0.538878193	5.06E-15
TCGA-STES(N=569)	0.671143889	9.84E-76	0.383928552	2.01E-21	0.561677141	1.28E-48
TCGA-SARC(N=258)	0.41233593	5.19E-12	0.336287844	3.07E-08	0.394435783	4.92E-11
TCGA-KIRP(N=285)	0.461070261	2.09E-16	0.402741276	1.54E-12	0.449402516	1.42E-15
TCGA-KIPAN(N=878)	0.607692214	9.37E-90	0.566595878	1.06E-75	0.622006017	3.78E-95
TCGA-STAD(N=388)	0.69312289	7.72E-57	0.357305504	3.98E-13	0.565369412	3.73E-34
TCGA-PRAD(N=495)	0.470879272	1.10E-28	0.295509951	1.97E-11	0.401444392	1.36E-20
TCGA-UCEC(N=178)	0.33525638	4.77E-06	0.219928284	0.003180421	0.302753795	4.00E-05
TCGA-HNSC(N=517)	0.533511932	2.28E-39	0.249831097	8.49E-09	0.422761103	7.88E-24
TCGA-KIRC(N=528)	0.431722039	2.20E-25	0.390797458	1.04E-20	0.470953941	1.67E-30
TCGA-LUSC(N=491)	0.637482639	2.36E-57	0.474293676	6.58E-29	0.578316561	3.64E-45
TCGA-THYM(N=118)	0.410627928	3.87E-06	0.001916236	0.983569462	0.237007153	0.009763974
TCGA-LIHC(N=363)	0.37364737	1.80E-13	0.410649646	3.36E-16	0.427722432	1.41E-17
TCGA-THCA(N=503)	0.437139185	6.86E-25	0.393283232	4.70E-20	0.435214517	1.16E-24
TCGA-MESO(N=85)	0.469366266	5.86E-06	0.309006872	0.004008421	0.424767134	5.08E-05
TCGA-READ(N=91)	0.695450199	2.02E-14	0.552058159	1.41E-08	0.657460371	1.45E-12
TCGA-SKCM-M(N=351)	0.554125441	1.22E-29	0.457065513	1.61E-19	0.529733993	8.76E-27
TCGA-SKCM(N=452)	0.514396229	6.74E-32	0.414106768	3.72E-20	0.48372772	6.92E-28
TCGA-PAAD(N=177)	0.715202855	4.98E-29	0.530636052	3.04E-14	0.658338555	2.34E-23
TCGA-OV(N=417)	0.698452859	2.82E-62	0.462585035	1.68E-23	0.618439484	2.30E-45
TCGA-TGCT(N=132)	0.604355207	1.69E-14	0.124143531	0.15612443	0.345343593	5.00E-05
TCGA-PCPG(N=177)	0.050377964	0.50546896	0.04063869	0.591230424	0.042769872	0.571908293
TCGA-SKCM-P(N=101)	0.307616823	0.001752372	0.202456574	0.042314353	0.269571802	0.006408718
TCGA-UVM(N=79)	0.23503819	0.037062679	0.325321795	0.003439484	0.311888819	0.005140382
TCGA-UCS(N=56)	0.44785351	0.000538004	0.226474507	0.093260929	0.357821459	0.006777549
TCGA-BLCA(N=405)	0.71898319	1.17E-65	0.549216903	2.75E-33	0.676473744	1.70E-55
TCGA-ACC(N=77)	0.355342573	0.001517584	0.312162201	0.005712621	0.324283538	0.004011933
TCGA-KICH(N=65)	0.433777096	0.00030643	0.391088219	0.001276501	0.420469038	0.000487918
TCGA-CHOL(N=36)	0.622028527	5.12E-05	0.472276019	0.003634281	0.559984423	0.000383214
TCGA-DLBC(N=46)	0.422724106	0.003425634	0.636438753	1.99E-06	0.670241823	3.48E-07
